# Temporal trends indicate an epidemiological shift in the pathology of mesial temporal lobe epilepsy

**DOI:** 10.1007/s00415-025-13398-1

**Published:** 2025-09-24

**Authors:** Christoph Helmstaedter, Sarah Al-Haj Mustafa, Juri-Alexander Witt

**Affiliations:** Department of Epileptology, University Hospital Bonn, Venusberg-Campus 1, 53127 Bonn, Germany

**Keywords:** Mesial temporal lobe epilepsy, Ammon´s horn sclerosis, Hippocampus, Limbic encephalitis, Temporal trends, Epidemiological shift

## Abstract

**Background:**

Recent multicenter studies suggest a temporal trend of a decreasing number of patients with classic early-onset mesial temporal lobe epilepsy (mTLE) with ammon’s horn sclerosis (mTLE–AHS). In parallel, the awareness of late-onset mTLE patients with suspected limbic encephalitis (sLE) is increasing. To evaluate a potential epidemiological shift, a large cohort of mTLE patients collected over 4 decades was analyzed.

**Methods:**

Anonymized data sets of a monocentric cohort of 1,556 patients with the diagnosis of mTLE, who underwent their very first neuropsychological assessment between 1986 and 2024 in the Department of Epileptology at the University Hospital Bonn in Germany, were retrospectively evaluated in regard to temporal trends of age, age at epilepsy onset, neuropsychological performance, and MRI pathology. Five-year intervals were evaluated.

**Results:**

Most significant was a trend of an increasing age at epilepsy onset over time (from 12 to 36 years), education and IQ increased over time, impairments became less global, and verbal/figural memory impairments became less marked and discriminative over time. While the number of patients with mTLE remained quite stable since 1996 (50–60% of all TLE patients seen), patients with early-onset mTLE–AHS (*n* = 1079, average age at epilepsy onset: 16 years) faded over time (from 100 to 32%), while the patients with late-onset mTLE suspicious of limbic encephalitis (*N* = 477, average age at epilepsy onset: 40 years) became increasingly prevalent (from 0 to 68%).

**Conclusions:**

Trends of changing etiologies as well as altering clinical and neuropsychological features of patients with mTLE suggest an epidemiological shift over the past decades. Overlapping distributions of fading early-onset classic mTLE–AHS and an increasing influx of late-onset sLE fit the clinical observations and demand retrospective follow-up studies in other countries/regions to disentangle triggering factors. Prospective studies should investigate temporal trends in autoantibody subgroups of patients with sLE.

## Introduction

Mesial temporal lobe epilepsy with ammon’s horn sclerosis (mTLE–AHS) has historically represented the most common form of drug-resistant focal epilepsy and has long been considered the prototypical surgical epilepsy syndrome. However, recent evidence suggests a notable temporal decline in the incidence and surgical treatment of this entity. Several multi-institutional and national-level studies have identified shifting patterns in the epidemiology, diagnostic approach, and surgical management of mTLE–AHS, raising important questions about the underlying causes and future implications of these changes.

Asadi-Pooya and colleagues estimated that the prevalence of drug-resistant mTLE–AHS in the United States ranges between 0.51 and 0.66 cases per 1,000 individuals, with an annual incidence of 3.1–3.4 per 100,000 [1]. Despite this substantial burden, a growing body of literature points to declining surgical intervention rates for this condition. Englot and colleagues, analyzing national inpatient data from 1990 to 2008, reported a paradoxical decrease in resective epilepsy surgeries, including anterior temporal lobectomies, even after the publication of Class I evidence supporting surgical treatment for drug-resistant epilepsy [7]. This trend was echoed by Kaiboriboon and colleagues, who found that anterior temporal lobectomies for mesial temporal sclerosis declined by over 65% between 2006 and 2010, while procedures for extratemporal and non-lesional epilepsies increased [19].

Data from specialized epilepsy centers corroborate these findings. In 2015, Jehi and colleagues documented a 48% decline in surgeries for mesial temporal sclerosis across nine major epilepsy centers between 1991 and 2011 [18]. Similarly, in 2016 Cloppenburg and colleagues reported a stable number of epilepsy surgeries over a 23 year period despite increasing presurgical evaluations, with a noticeable decrease in surgeries for medial temporal sclerosis after 2009 [6]. This may reflect a growing proportion of patients with ambiguous diagnostic findings, a higher rate of rejection of surgery by the patient, or a shift towards more conservative and palliative treatment approaches.

From a histopathological standpoint, Blumcke and colleagues, in an extensive analysis of over 9,500 surgical specimens across Europe, found that while hippocampal sclerosis remained the most common pathology in adults, its relative frequency varied substantially by center and appeared to decline over time [3]. In Germany, our group reported in 2014 that although hippocampal sclerosis remained the predominant pathology among patients undergoing temporal lobe surgery between 1988 and 2008, the average age at surgery and the epilepsy duration increased significantly among mTLE–AHS patients, suggesting a decreasing incidence or delayed referral pattern [15]. At the same time an increasing number of late-onset mTLE, many of them suspected to suffer from limbic encephalitis, was noted.

International data further support these observations. In the UK, Neligan and colleagues observed a decline in all resective epilepsy surgeries between 2000 and 2011, except for vagus nerve stimulator implantations, with temporal lobe resections still being the most common but declining in frequency [26]. In a U.S. population-based analysis, Kwon and colleagues confirmed a statistically significant decline in lobectomy and amygdalohippocampectomy rates from 2003 to 2014, raising additional concerns regarding underutilization of surgery [21].

Collectively, these studies point to a consistent trend: the number of patients with classical drug-resistant mTLE–AHS undergoing surgery has declined over the past two to three decades, despite expanding diagnostic capabilities and increasing awareness of the benefits of early surgical intervention. Whether this reflects a true epidemiological shift—potentially due to changing environmental or perinatal risk factors—or evolving treatment paradigms and patient preferences remains a topic of active investigation.

In this context, another clinical trend appears to be of major importance. Alongside the decline in early mTLE–AHS cases, there has been increasing recognition of late-onset mTLE in the context of inflammatory and autoimmune epilepsies. At our center, this awareness began around 2000 [2] with isolated cases of patients and became routine between 2010 and 2020 [9, 14, 22, 23, 28]. In 2004 an ILAE Commission report declared mTLE–AHS a cluster of signs and symptoms to make up a syndromic diagnostic entity [35]. At that time, patients with mTLE suspected of having limbic encephalitis were not recognized as a significant group. Furthermore, while many studies addressed mTLE surgery in elderly subjects, the issue of late-onset mTLE beyond the age of 50 years had not been explicitly discussed—perhaps because the syndrome was originally defined based on a patient population in which epilepsy typically began early in life, or, as hypothesized here, because patients with later onset were simply not present at that time. This situation contrasts sharply with findings from a 2020 report on adult-onset temporal lobe epilepsies. Eight hundred adult-onset non-lesional temporal lobe epilepsy patients had been consecutively seen between 2013 and 2016 in our center in Bonn, Germany. Thirteen percent of these 800 patients had been tested positive for neural antibodies, and an additional 25% had been screened positive using indirect immunofluorescence on hippocampal/cerebellar sections and immunoblots of whole-brain and synaptosome lysates [20]. Both the high incidence of late-onset mTLE in that short period of time and the large number of patients with suspected autoimmune etiology are striking. A study which tested for antibodies in chronic long-term epilepsies in contrast revealed only very few positive patients (0.4% of 223 patients) [34]. The pressing question is whether this group of late-onset mTLE with suspected limbic encephalitis was previously overlooked or whether we are witnessing the emergence of a new clinical phenomenon. If the latter is indeed the case, the situation mirrors that of mTLE–AHS, where an explanation is needed for the syndrome’s appearance and increasing disappearance leading to the observed epidemiological and pathological shift.

Taking this as the basis, the present study aims to bridge the gap between the declining trend of mTLE–AHS and the emergence of late-onset TLE patients suspected of having inflammatory or autoimmune conditions. It includes all patients with diagnosed mTLE seen in the neuropsychological unit of the level 4 epilepsy center at the University Hospital Bonn between 1986 and 2024.

## Materials and methods

### Patients

In the present monocentric study, we included 1556 temporal lobe epilepsy patients with the diagnosis of mesial temporal lobe epilepsy (mTLE) who had been submitted for a neuropsychological (often presurgical) assessment to the neuropsychological unit of the level 4 epilepsy center at the University Hospital Bonn during the years 1986–2024. This group represents 51.2% of all patients with the diagnosis of temporal lobe epilepsy (*n* = 2,784) screened for the first time during that period. All patients suffered from epileptic seizures.

Of the 1556 patients with mTLE, 47% were female, 63% had an education of > 9 years, the average chronological age was 41.0 ± 14.4 years, the average age at epilepsy onset was 23.8 ± 19.7 years, and the average duration of epilepsy was 17.3 ± 14.9 years.

Of all mTLE patients, 1,079 (69.3%) had mTLE with ammon’s horn sclerosis (AHS), and 477 (30.7%) fulfilled the clinical criteria to suspect limbic encephalitis according to Graus [11].

### Evaluating temporal trends

For the evaluation of temporal trends, the covered time period from 1986 to 2024 was split into 5 year intervals and one 4 year interval (2021–2024). Clinical and neuropsychological parameters of interest were pathology, i.e., mTLE–AHS vs. suspected limbic encephalitis (sLE), chronological age, age at epilepsy onset, lateralized MRI findings, education, intelligence (vocabulary IQ), neuropsychological phenotypes as defined by the so called IC-CoDE (International Classification [17, 25]).

### Data availability

The data supporting the findings of this study are available from the corresponding author upon reasonable request.

## Results

### Incidence of mTLE in TLE over time

**Fig. 1:**
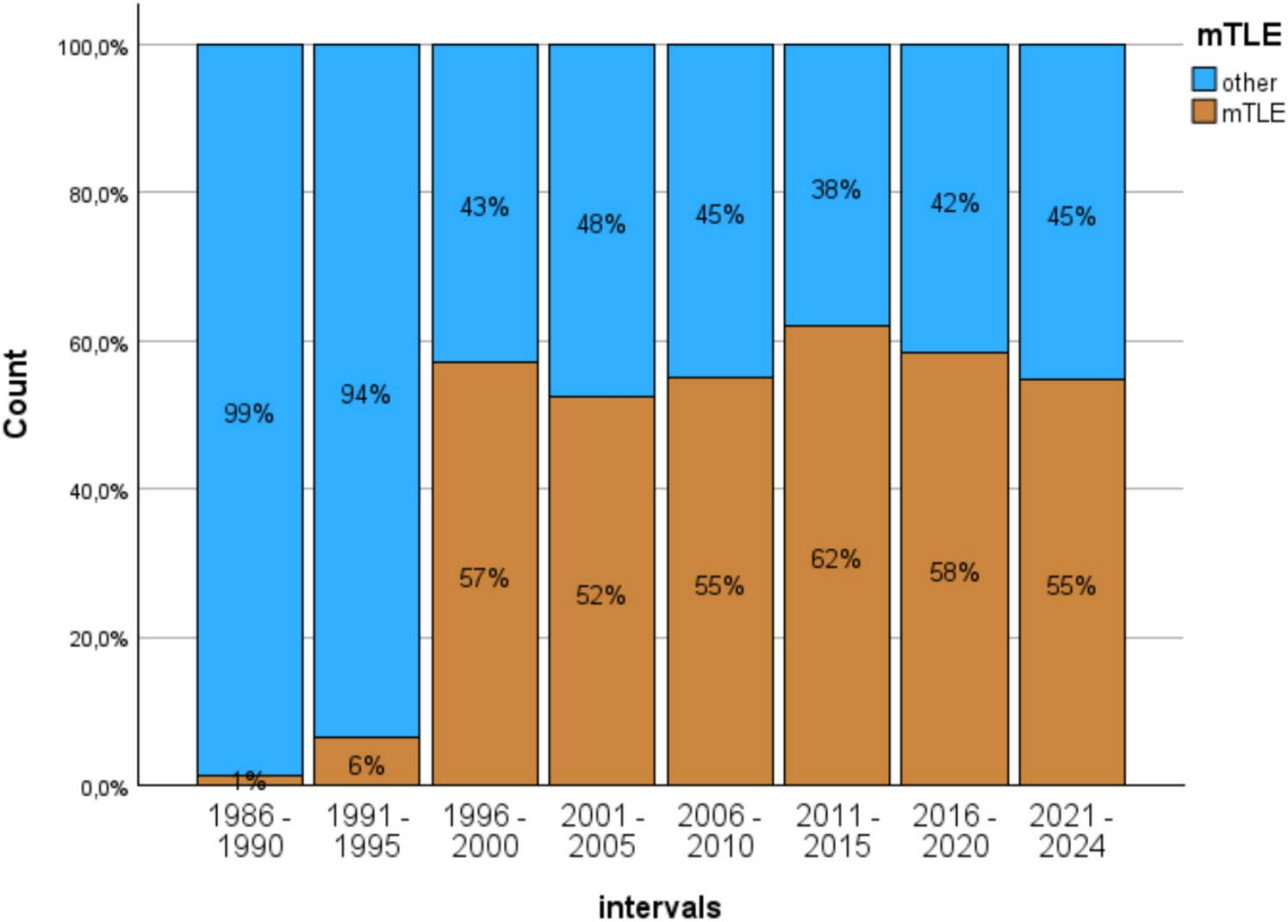
Incidence of mTLE in TLE over time. The diagram shows the proportion of patients with mTLE (*N* = 1556) in the group of all patients with TLE (*N* = 2784) seen in the neuropsychological unit at the Department of Epileptology in Bonn over time

Taking all 2,784 TLE patients seen in the observation interval as the basis, mTLE was diagnosed in 85 patients in 1986–1990, in 202 in 1991–1995, in 484 in 1996–2000, in 501 in 2001–2005, in 355 in 2006–2010, in 350 in 2011–2015, in 507 in 2016–2020, and in 300 in 2021–24. Thus, there is a steep increase in the numbers from 1 to 6% until 1995 to 50–60% in the time intervals to follow (χ²(7) = 284.3; *p* < 0.001, see Fig. 1).

### Increasing age at epilepsy onset in mTLE

**Fig. 2:**
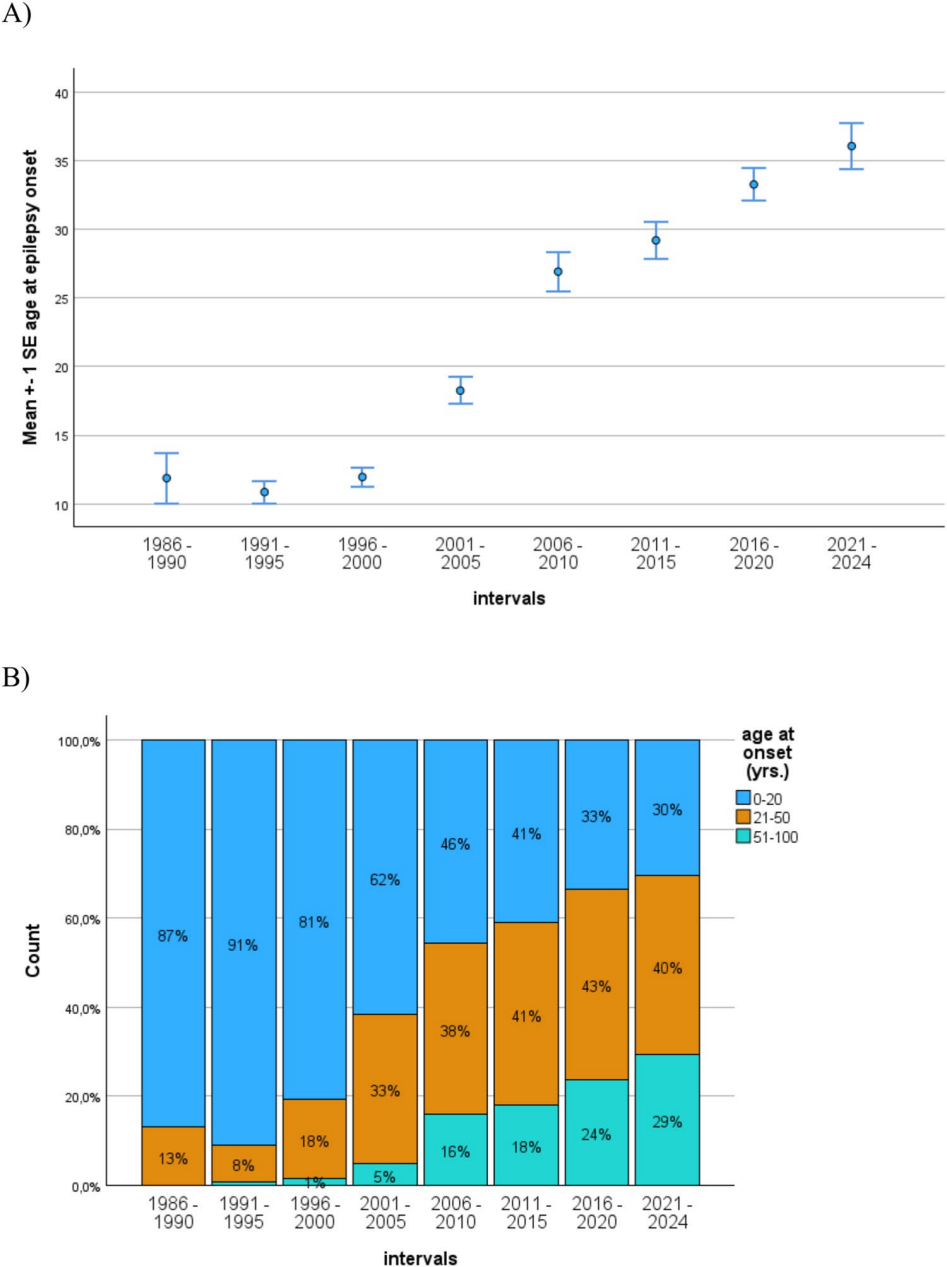
Increase of age at epilepsy over time. **A** The average age at epilepsy onset increased from about 11–12 years in the three first time periods to 18 years in 2001–2005 and 36 years in 2021–2024 **B** Categorizing the age at the onset of epilepsy into early intermediate and late, major changes across the time intervals become evident

Restricting the analysis to patients with mTLE (*N* = 1556), there is strong evidence that the age at epilepsy onset stays stable at an average of 11–12 years between 1986–2000 before increasing considerably in the time after, reaching a mean of 36.0 years in the final period 2021–2024. Thus, age at onset approximately tripled over time. (ANOVA: *F* = 60.5, *p* < 0.001, *η*^2^ = 0.215; see Fig. 2a).

Correspondingly, the patients’ duration of epilepsy steadily decreased from 20 ± 11 and 22 ± 12 years in the period 1986–1995 to 12 ± 14 and 10 ± 14 years in 2016–2024 (*F* = 31.3; *p* < 0.001, *η*^2^ = 0.12).

Chronological age steadily increased as well from 32 ± 8 and 33 ± 9 years in 1986–1995 to 45 ± 16 and 46 ± 17 years in 2016–2024 (*F* = 19.4; *p* < 0.001, *η*^2^ = 0.08).

Categorizing the age at epilepsy onset into early, intermediate, and late-onset epilepsies, we applied a scheme of an onset before age 21 covering the time of mental development brain maturation, the time from 21 to 50, and the time later than 50, when the brain’s disintegration and ageing become increasingly evident (see Fig. 2b**).**

The figure indicates major changes in the proportions, particularly of early (age 0–20) and late-onset epilepsies (> age 50) over time. There were almost no patients with an onset later than 50 years of age until 2005 (0–5%); between 2006 and 2024 the numbers rise from 16 to 29%. Vice versa between 80 and 90% of the patients had an early onset before age 20 in the period until 2000. The number declined thereafter from 62% to finally 30%. The intermediate group with onsets from age 21 until age 50 increased from 13 to 33% in the period until 2005. Thereafter, stable proportions around 40% are observed (χ²(14) = 307; *p* < 0.001).

### Increasing bilateral MRI findings in mTLE

**Fig. 3:**
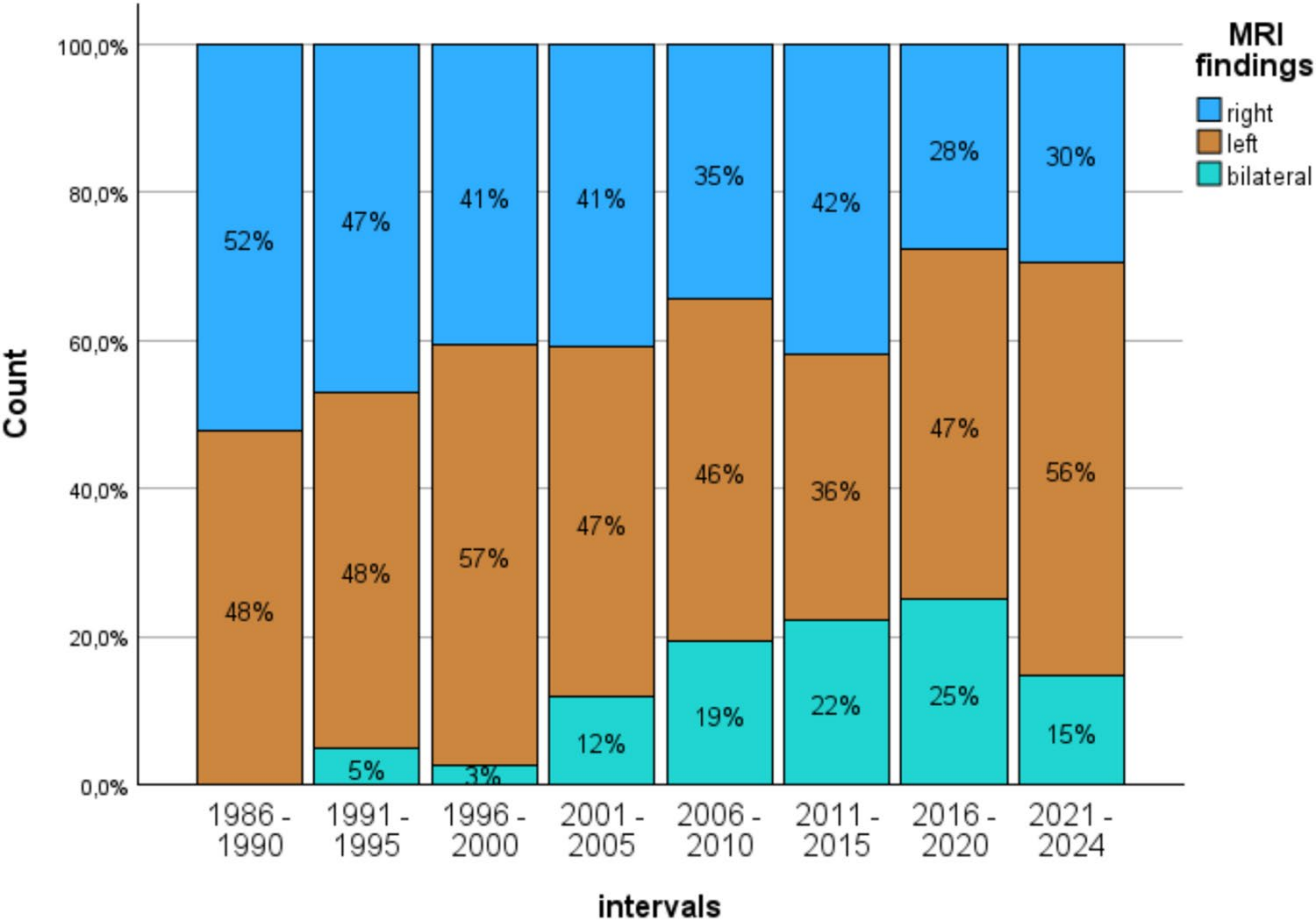
Increase of bilateral pathologies over time. Imaging data indicate a significant increase of bilateral pathological findings between 2001 and 2020

MRI data, which were available for 1432 patients, indicate a steady increase of bilateral pathologies over time until 2020, starting with zero patients in 1986–1990 increasing to 25% in 2016–2020 and 15% in 2021–2024 (χ²(7)= 98.4; *p* < 0.001, see Fig. 3).

### Changes of neuropsychological features over time in mTLE

**Fig. 4:**
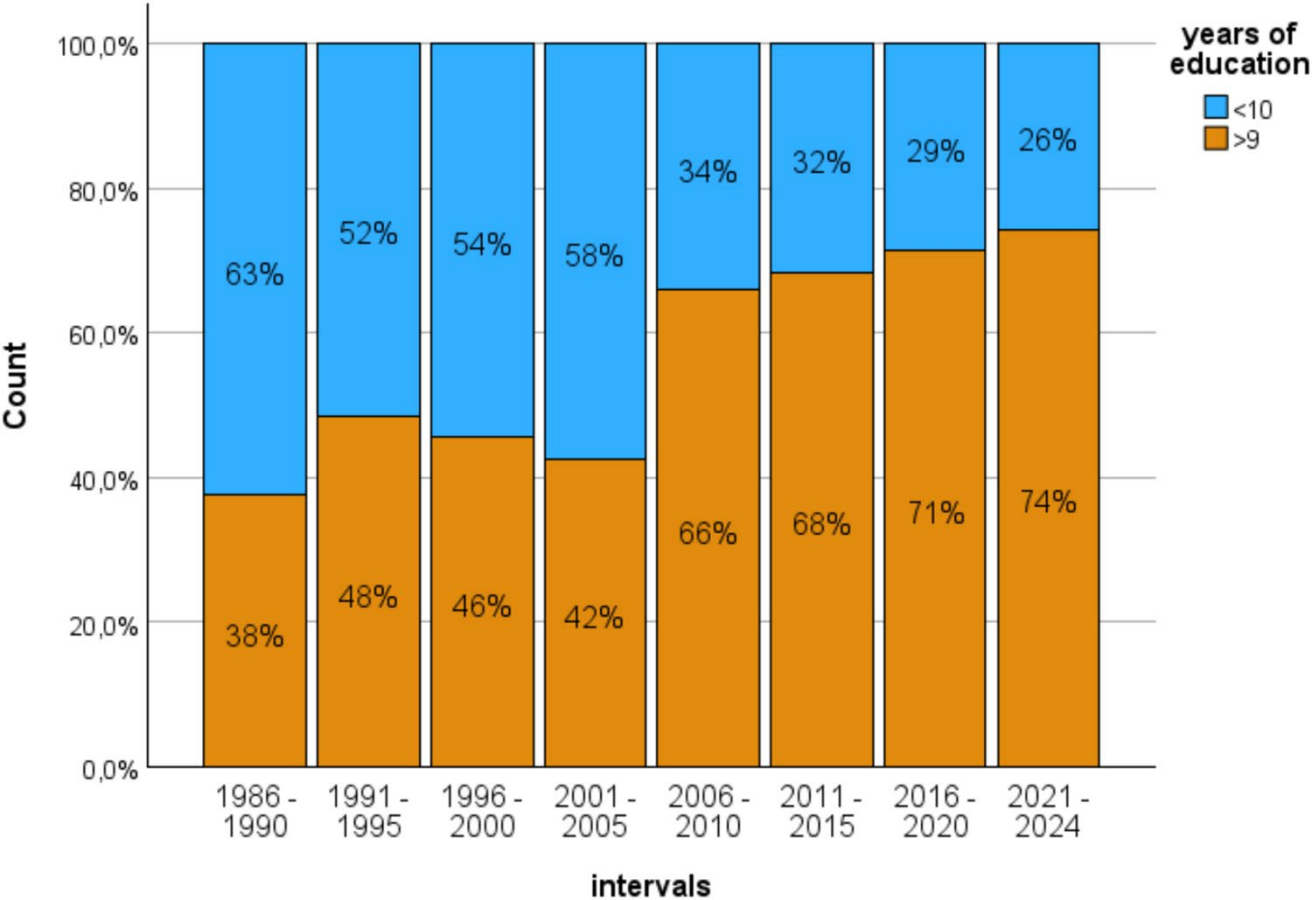
Increase of years of education over time. The number of patients completing more than 9years of education almost doubles over time from 38 to 74%

IQ data, which were available for 1003 patients, indicate a weak but steady and significant increase from 95 in 1986–1990 to 108 in 2021–2024 (*F* = 7.4; *p* < 0.001, *η*^2^ = 0.05). This trend is even better reflected by the years of education. The number of patients with more than 9 years of education steadily increased from 38% in 1986–1990 to 74% in 2021–2024 (χ²(7)= 56.5; *p* < 0.001, see Fig. 4).

Using the so-called IC-CoDE as a simple method to retrospectively categorize and review performances across multiple cognitive domains in addition to IQ, 1,473 mTLE patients with sufficient domains tested were categorized as to whether they show no impairment, impairment in one domain, two domains, or more than two domains.

**Fig. 5:**
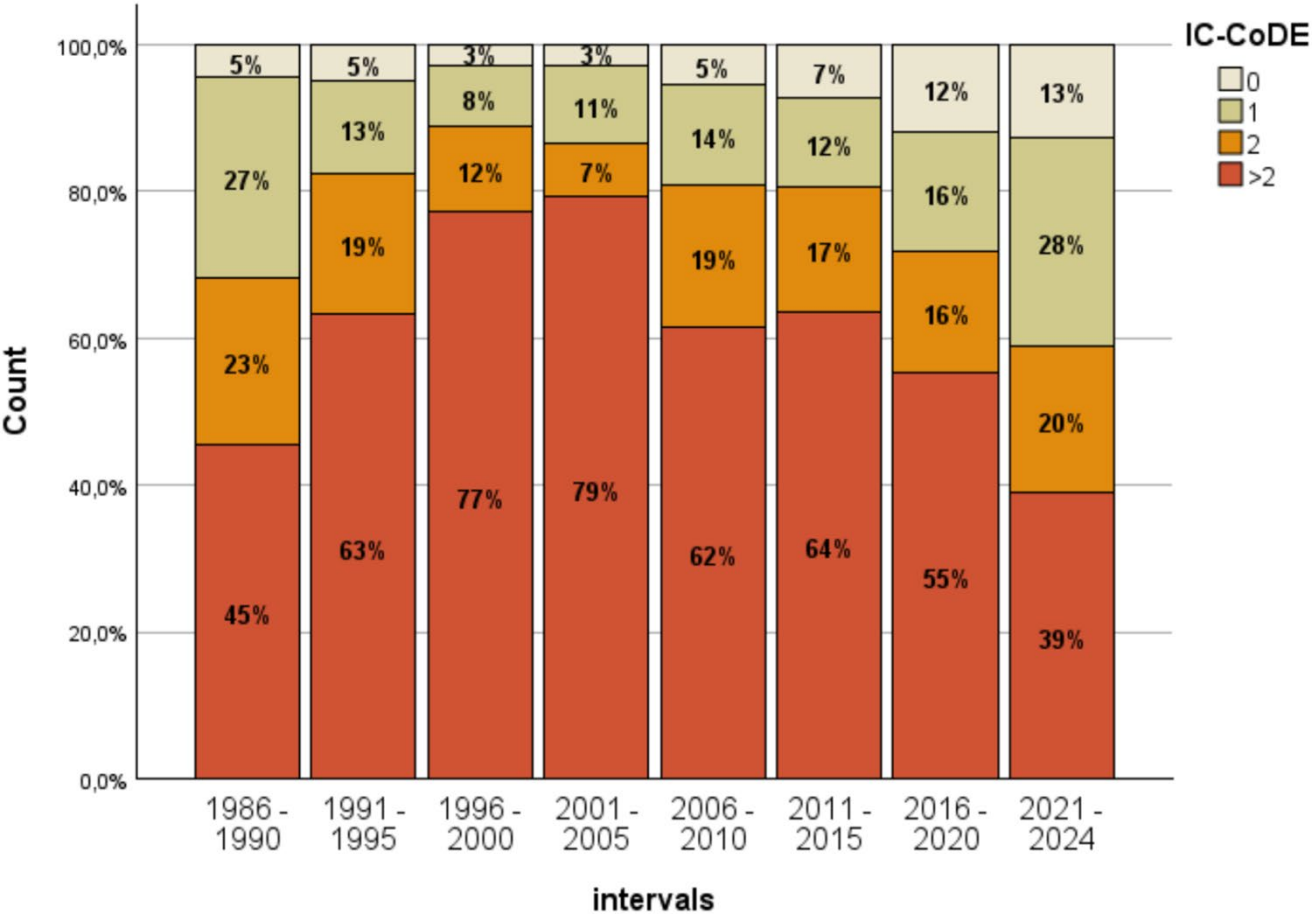
Decrease of the range of impairments over time. Applying a simplified cognitive performance categorization (IC-CoDE), first an increase of broader impairments is seen between 1986 and 2005 followed by a trend of increasingly more patients with no or only one domain impaired until 2024

As indicated by Fig. 5 cognitively broad impairment (> 2 domains impaired) increased from 45 to 79% in the time interval until 2001–2005 and then steadily decreased to 39% in the interval 2021–2024 (χ²(21)= 120.5; *p* < 0.001). Going along with this, particularly the groups with no or only one domain impaired changed more than those with two domains impaired.

Going into more detail, the data in regard to verbal and figural memory (results were categorized as: 0 = severely impaired, 1 = impaired, 2 = borderline, 3 = average, 4 = above average, one point corresponding to about one standard deviation) show that the specificity for indicating a lateralized material-specific deficit fitting to the side of the lesion differs depending on whether left/right differences are calculated or whether verbal and figural memory are related to each other in left and right temporal lobe epilepsy patients separately.

**Fig. 6:**
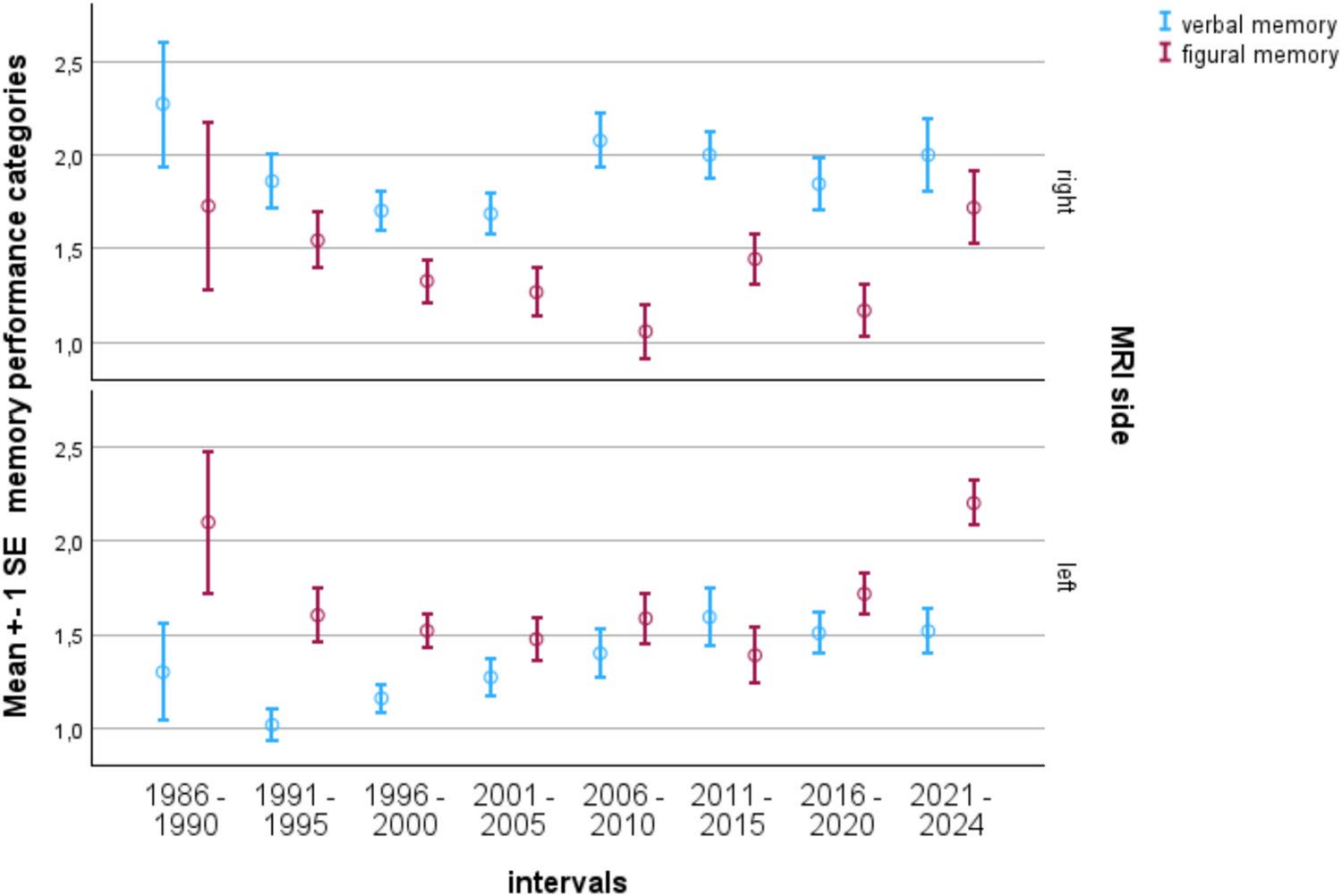
Material-specific memory as a function of time and lateralization of epilepsy. Verbal memory better differentiates between lateralized epilepsies, figural memory better within the lateralization groups

The between group analysis shows that while left mTLE patients are consistently worse than right mTLE patients (F between 5.1 and 25.6, *p* < 0.05–0.001), figural memory differentiates only in the periods 2006–2010 and after 2016 (*F* between 4.4 and 9.0, *p* < 0.05–0.001; Fig. 6).

Relating verbal and figural memory to each other within the left and the right mTLE group, the “verbal memory worse than figural memory” pattern in left mTLE (*N* = 533) changed over time whereas the pattern “figural memory worse than verbal memory” appears quite stable in right mTLE (*N* = 694). In right mTLE, the effect verbal vs. figural memory was highly significant with *F* = 50.7, *p* < 0.001, but no significant interaction with the time interval was observed. In left mTLE we observed a significant effect regarding verbal vs. figural memory (*F* = 27.0, *p* < 0.001), as well as a significant interaction effect with the time interval (*F* = 3.4, *p* < 0.01). As depicted in Fig. 6, there are time intervals (2006–2020) in the left temporal group in which their verbal memory is hardly worse than figural memory. Interestingly, in the same time intervals, the differences between verbal and figural memory are strongest in the right temporal group.

Thus both viewpoints provide evidence that left temporal lobe patients do worse on verbal memory than right temporal lobe patients, that figural memory mostly does not differentiate between the groups, but that within the groups figural memory is consistently worse than verbal memory in right temporal lobe patients whereas there are periods during which verbal memory is not poorer than figural memory in left temporal lobe patients.

### mTLE–AHS vs. sLE

An explanation for these findings is provided when splitting the total group of mTLE into mTLE–AHS patients (*nN *= 1079) vs. patients with mTLE and suspected limbic encephalitis (sLE) (*nN* = 477) and when looking at their occurrence over time as well as their clinical and neuropsychological differences.

**Fig. 7:**
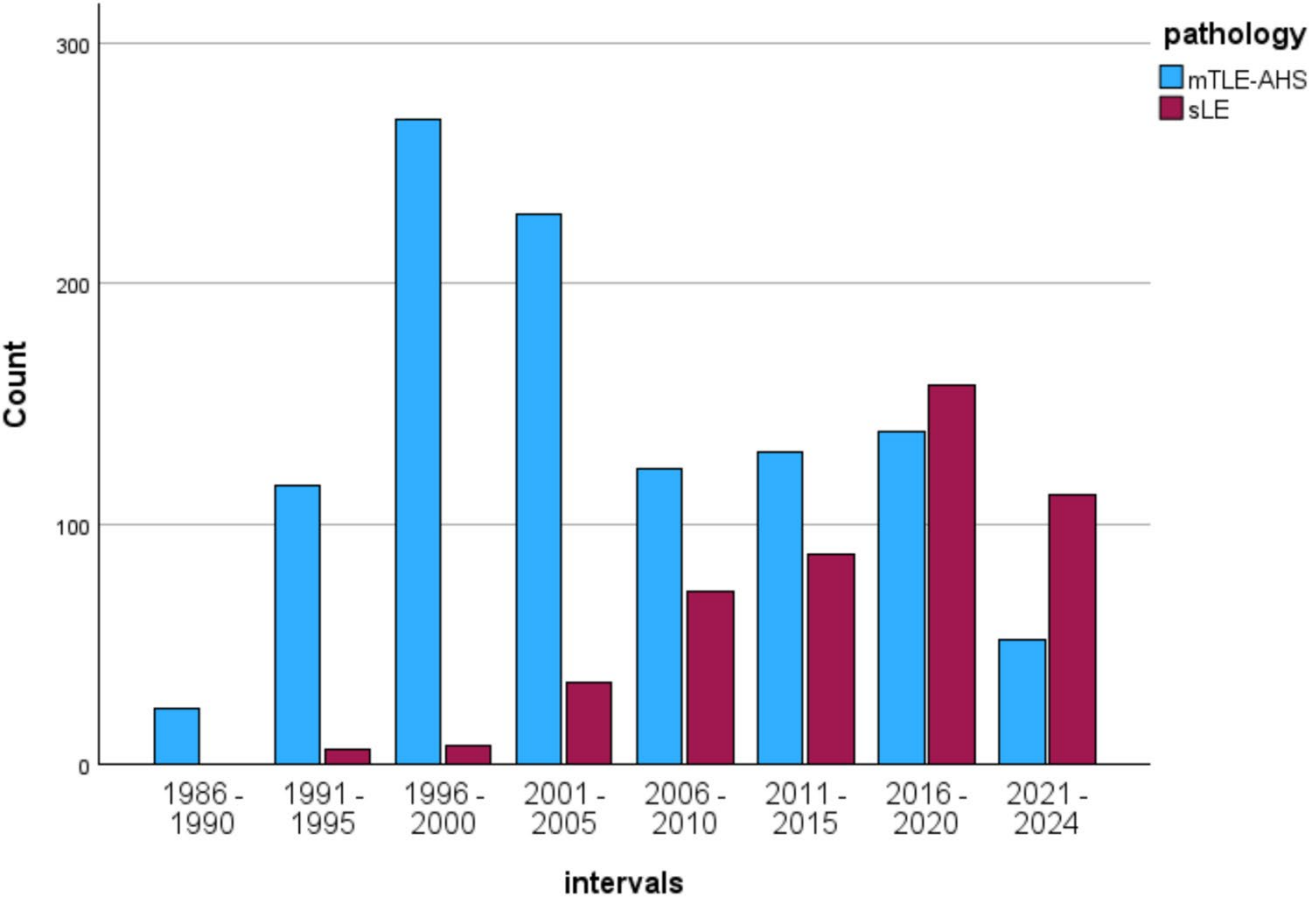
Overlapping distributions of patients mTLE–AHS and sLE. The bar chart demonstrates the overlap of the increase of mTLE–AHS patients until 2005 and their decrease until 2024 with a steady increase of patients with sLE

As shown in Fig. 7**,** two overlapping cohorts become evident over time with patients with mTLE–AHS being increasingly replaced by those with sLE. The percentage of patients with sLE starts with zero patients in 1986–1990, rises from 12.9% in 2001–2005 to 36.9% in 2006–2010 and increases to 68.3% in 2021–2024 (χ²(7) = 380.9; *p* < 0.001).

The groups do not differ in regard to gender (mTLE–AHS 48% female, sLE 45%) and only marginally in regard to age, sLE patients being older than mTLE–AHS patients (mTLE–AHS 39.1 ± 12.9 years, sLE 45.6 ± 16.6 years, F = 71.2, *p* < 0.001, η^2^ = 0.04).

The groups do, however, differ highly significantly in regard to the age at epilepsy onset (mTLE–AHS 16.6 ± 15.2 vs. sLE 40.1 ± 18.8 years, F = 673.2, *p* < 0.001, η^2^ = 0.30) and the duration of epilepsy (mTLE–AHS 22.5 ± 13.9 vs. sLE 5.5 ± 9.5 years, F = 588.9, *p* < 0.001, η^2^ = 0.27).On average, the disease occurs more than 20 years later in patients with sLE, while patients with mTLE-AHS have a disease duration that is approximately four times longer.

Imaging more frequently revealed bilateral pathologies in sLE patients (mTLE–AHS 10.5% vs. sLE 24.7%, χ²(1)= 47.4; *p* < 0.001).

Furthermore, the groups differ in regard to IQ (mTLE–AHS 101.2 ± 13.5 vs. sLE 108.5 ± 14.6, F = 59.4; *p* < 0.001, η^2^ = 0.06), education (mTLE–AHS 55.2% > 9 yrs. vs. sLE 76.0%, χ²(1)= 50.8; *p* < 0.001), and IC-CoDE categorization (mTLE–AHS 4% with no impairment and 73% with greater two domains impaired vs., sLE 14% unimpaired and 44% with broader impairment (χ²(3) = 131; *p* < 0.001). Patients with sLE are more intelligent, they are better educated, and they are less broadly, i.e. more partially impaired.

**Fig. 8:**
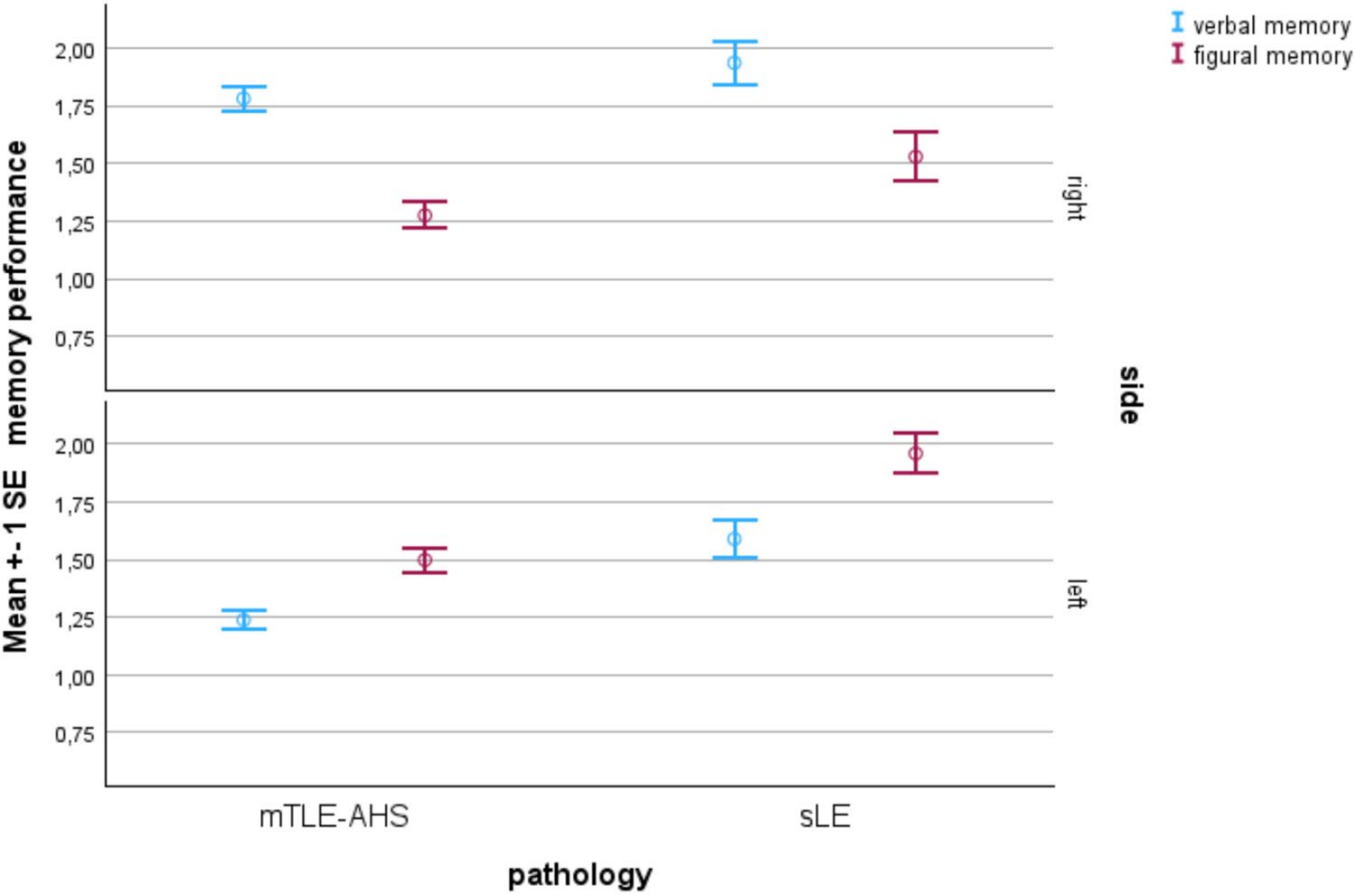
Material-specific memory as a function of the lateralization and pathology. Verbal and figural memory differentiate between and within lateralized mTLE but statistical significance is achieved only in mTLE–AHS

Within the lateralization groups, the pattern of “verbal worse than figural memory” and of “figural worse than verbal memory” was associated with the lateralisation of mTLE–AHS (interaction effect F = 31.3; *p* < 0.001). This was not the case for patients with sLE (F = 2.5; *p* = 0.12). Between the groups, patients with sLE performed significantly better in both memory domains when compared to patients with mTLE–AHS (verbal: mTLE–AHS 1.4 ± 1.0 vs. sLE 1.6 ± 1.1, F = 19.4; *p* < 0.001, η^2^ = 0.01; figural: mTLE–AHS 1.4 ± 1.1 vs. sLE 1.8 ± 1.2, F = 41.3; *p* < 0.001, η^2^ = 0.03; Fig. 8).

### Early- vs. late-onset mTLE

As evident from the presented data above, the clinical differentiation of mTLE–AHS and sLE and the age at epilepsy onset are highly confounded. Two different syndromatic pathologies are hypothesized, though their definitive diagnostic delineation remains relative.. Figure 2b already indicates that the temporal trend is mostly seen in the two groups with an epilepsy onset before age 21 vs. beyond age 50.

A cross tabulation of the age at onset vs. the study groups demonstrates that 95% of the mTLE–AHS patients have an onset before age 50, and 70% before age 21, whereas 82% of those with suspected LE start after age 21 and 32% after 51 (χ²(2) = 416.1; *p* < 0.001).

Therefore, the analyses from the last paragraph were repeated for more strictly separated groups selecting only mTLE–AHS starting before 21, and sLE starting after 20. The results are not presented since the global picture was almost the same.

## Discussion

Clinical observations at a level 4 university epilepsy center with a comprehensive surgical program suggest that the population of patients with TLE is changing over time. Surgical case numbers are declining, and there appear to be fewer patients with classic early-onset mTLE. At the same time, there is a growing influx of patients with late-onset mTLE—often non-lesional (i.e., without tumors, developmental anomalies, cerebrovascular disease, or post-traumatic pathology)—many of whom are suspected to suffer from limbic encephalitis [29].

That the landscape is indeed changing is supported by several recent studies of multicenter surgical cohorts [5–7, 15, 18, 19, 21, 26] and this is strikingly confirmed by findings from the present comparably large monocentric cohort, collected over close to four decades in the neuropsychological unit of the Department of Epileptology at the University Hospital Bonn, Germany.

Discussing these results, it should be stated from the outset that the reported cohort—2,784 TLE patients, of whom 1,556 were diagnosed with mTLE—is selective, and representative only of patients with suspected TLE who underwent neuropsychological evaluation early during their stay at a highly specialized epilepsy center, often as part of a presurgical assessment. The selectivity of the diagnostic distinctions mTLE–AHS and sLE is a matter of debate, but the large sample size may compensate for some degree of diagnostic imprecision. Because the cohort spans such a long time interval, emerging trends become visible that otherwise would be difficult to detect. These trends challenge our understanding of mTLE, its underlying pathologies and potential etiological factors, and its treatment.

The results of the first part of the evaluation suggest that the diagnosis of mTLE, i.e. seizures originating from mesial temporal lobe structures, is quite stable since 1996 with between 50 and 60% of the TLE patients seen. The change from 1986 to 1996 may indicate an increase of mTLE but should more likely be interpreted considering that imaging improved considerably in this time period, increasing the hit rate of underlying pathologies. For example, lesion detection in MRI scans of 123 patients at our center increased from 39% when read by “non-expert radiologists” to 50% when read by “expert radiologists” and finally to 91% when epilepsy dedicated imaging was performed [32].

However, while the rate of patients with mTLE appears stable between 2016 and 2024, the patients’ demographic and clinical characteristics indicate in part highly significant temporal trends. The average chronological age of the patients increased by about 10 years, the duration of epilepsy halved from approximately 20 to 10 years, the age at epilepsy onset tripled from circa 12 to 36 years, and bilateral MRI findings increased from under 10 to 25%. In addition, the cognitive data indicate that the number of patients with better education almost doubled over time from 41 to 76%, IQ steadily increased up to about 10 points. Broader impairments first increased from 45% in 1986 to close to 80% in 1996–2005 and decreased thereafter to currently 39%.

Since episodic memory is the most vulnerable cognitive domain in TLE and mTLE, particular attention was given to verbal and figural memory—both in absolute terms and relative to each other—taking epilepsy lateralization into account.

Consistent with the literature, verbal memory differentiated left and right lateralized epilepsies better than figural memory most of the time. However, the within-group analyses showed that in right mTLE figural memory was consistently worse than verbal memory as we have reported before, while verbal memory compared to figural memory was increasingly indifferent in left mTLE. The difference was solid in the years until 2000, this being the time when we published most of our results on material-specific memory impairment in lateralized TLE [10, 12, 13, 16, 31].

Assuming that the tests’ validity will not change over time, one is inclined to suggest that the epilepsies and their underlying pathology might have changed. With the changes in cognition and cognitive patterns, we probably observe one clinical correlate (pathological groups; early- vs. late-onset epilepsy) of what has been proposed as neuropsychological phenotypes in epilepsy when applying the IC-CoDE in temporal lobe epilepsy [25, 30].

This leads over to the second part of the evaluation which fills the gap between the years we profited of the uniform mTLE–AHS in a surgical context and the current era, in which autoimmune limbic encephalitis has become a prominent issue.

Figure 7 impressively depicts how the frequency distributions of patients with mTLE–AHS and sLE overlap and how the groups begin to replace each other in the late 1990s. While at the beginning there were no cases with sLE, this group is clearly dominating in the last observation period with 68%. On average, the group of sLE patients is 7 years older, the epilepsy onset is about 20 years later, and the duration of epilepsy four times shorter than in patients with mTLE–AHS. In addition, the sLE group more often has less lateralized MRI findings, they are more intelligent, better educated and less broadly, i.e. more partially impaired or even unimpaired, and the patients with sLE show better memory performances than those with mTLE–AHS. On a different performance level, the relation of verbal and figural memory to each other and to the lateralization of the epilepsy still indicates the lesion side in both groups, but the differences only reached significance in the mTLE–AHS group. This would match our observation that in patients with sLE memory and lateralization of the epilepsy are less reliable than in mTLE–AHS patients [36].

The major difference between sLE and mTLE–AHS is their different age at epilepsy onset. Temporal trends are mainly indicated for the extrema, i.e. early versus late onset, and less for the intermediate interval of epilepsies with onsets between 20 and 50. MTLE–AHS appears more strongly connected to an early onset until 20, while sLE is stronger connected to an onset beyond 50. The intermediate range is shared by both groups. Whether this reflects a diagnostic blurring and indicates that the etiologically distinct groups are to be found in the extrema cannot be determined within this dataset. Patients with mTLE in the intermediate group may have another triggering factor than those with an early onset. Moreover, patients with sLE in the intermediate group may have different immune responses than those in the late group. In one of our own studies, the two largest autoantibody groups seen in mTLE, i.e. patients with anti-GAD-65 (glutamate decarboxylase) and anti-VGKC (voltage-gated potassium channels) differed significantly regarding their age at onset, the VGKC group being on average 20 years older than the GAD group [9]. Future studies should investigate this issue and evaluate whether there are temporal trends particularly in the VGKC group.

In summary, the results strongly suggest that the underlying pathology in mTLE has changed over time. At least for mTLE–AHS a finite cohort of patients can be assumed. These had been ideal candidates for epilepsy surgery in the past most likely because their epileptic condition with hippocampal sclerosis was the residual defect of a disease process which lies in the past, the causes of which are still not well understood. Thus, one can postulate that the AHS is acquired and predominantly associated with an initial precipitating event [8, 24, 27].

As discussed in our previous work, reasons for the fading of mTLE–AHS may be a better treatment of bacterial and viral infections, successful treatment of febrile convulsions, vaccination programs, better seizure control or even antiepileptogenic effects of newer ASM [9]. Different from the condition in mTLE–AHS, patients with sLE represent a group with heterogeneous known, suspected and not yet known autoimmune origins: The underlying disease condition may still be active, it may lead to AHS, without knowing what has caused the condition and whether conceptual parallels to the origins of early-onset mTLE–AHS can be drawn. As it can be speculated for mTLE–AHS in retrospective, one may as well speculate that late-onset mTLE with sLE are triggered by endemic viral infections (e.g. via molecular mimicry). MTLE, however, persists as a symptom or residuum of obviously very different etiological factors which appear to systematically change over time. In this regard, the results indicate an epidemiological shift which will have consequences for the understanding of epilepsy, diagnostic requirements and treatment approaches. This change is in particular challenging for epilepsy surgery that might have to reconsider all concepts in regard to the prediction of the risks and benefits of epilepsy surgery and different invasive approaches which are based on results of a bygone cohort of patients with mTLE–AHS.

Follow-up studies would need to evaluate whether similar shifts are seen in other countries/regions as well, if so what their timing is, and whether this provides clues to understand the driving factors. For patients with mTLE–AHS this should be easier than for sLE patients, who appear heterogenous in regard to the presence/absence of antibodies and their antigenic target structure in particular. Whether the current phase of late-onset TLE patients with sLE will fade out later like it is seen with mTLE–AHS and whether disease conditions can be discerned which trigger autoimmune epilepsies needs to be determined in the future for specific antibody subgroups.

As already indicated above, this study has several shortcomings since it is the retrospective analysis of a monocentric neuropsychological database which has been maintained for nearly 40 years in a level 4 university epilepsy center with a comprehensive surgical program. The database was anonymized for this evaluation and has limited clinical data, primarily focused on aspects relevant to neuropsychological questions in epilepsy. Our study involving 800 patients collected in three years and published in 2020 [20] demonstrated that more patients pass through the clinic than are seen by neuropsychologists. The same study, however, demonstrated as well that 38% of these 800 patients were antibody or screening positive for an autoimmune etiology. Autoantibody negative sLE patients share clinical characteristics with positive patients and one should keep in mind that new antibodies wait to be detected [4, 14, 28, 33].

Several other potential biases warrant discussion. First, the absence of serological and cerebrospinal fluid data may have led to an overdiagnosis of suspected autoimmune limbic encephalitis (sLE). Second, the Graus 2016 [11] criteria for sLE were not applied to earlier cases. Finally, the findings are representative only of patients who underwent neuropsychological testing.

In this context, it is noteworthy that the almost complete absence of old and late-onset mTLE patients in the past is surprising, even in the absence of autoimmune diagnostics. One would expect such cases to have been identified in a university epilepsy clinic with over 7000 annual outpatient contacts, where all patients with evident or suspected TLE were screened for potential epilepsy surgery. While the Graus criteria were naturally not used prior to their publication in 2016, it is important to recognize that the clinical characterization of limbic encephalitis emerged concurrently with the increasing recognition of this patient group. Their prior absence can itself be seen as an indicator that a novel disease entity had entered the epilepsy landscape. The data presented here support this conclusion. It should be emphasized that this study is representative only of patients who, due to known or suspected TLE, underwent a formal neuropsychological evaluation as part of their pre-surgical workup. With approximately 25,000 neuropsychological assessments conducted since 1986, this evaluation was an integral and non-elective component of standard patient care in Bonn.

Leaving questions which require more detailed clinical data aside, the trends demonstrated in this currently unique cohort indicate an epidemiological shift in mTLE. This should initiate follow-up studies in countries with different health systems and conditions.
